# Sickle cell disease: embedding patient participation into an international conference can transform the role of lived experience

**DOI:** 10.1186/s13023-023-02951-8

**Published:** 2023-11-01

**Authors:** Mariangela Pellegrini, Subarna Chakravorty, Maria del Mar Manu Pereira, Beatrice Gulbis, Catriona Gilmour-Hamilton, Sandy Hayes, Mariane de Montalembert, Baba Psalm Duniya Inusa, Raffaella Colombatti, Noémi BA Roy

**Affiliations:** 1 European Reference Network on Rare Hematological Disease, the ERN-EuroBloodNets, Hôpitaux de Paris, Hôpital Saint Louis, Paris, France; 2 King’s College Hospital NHS Foundation Trust, London, UK; 3 European Reference Network on Rare Hematological Diseases, the ERN-EuroBloodNet, Vall d’Hebron Research Institute/Vall d’Hebron University Hospital, Barcelona, Spain; 4 European Reference Network on Rare Hematological Diseases, the ERN-EuroBloodNet, Hôpital Erasme/LHUB-ULB, Brussels, Belgium; 5 Oxford University Hospitals NHS Trust, Oxford, England; 6 European Reference Network On Rare Hematological Diseases, the ERN-EuroBloodNet, Hôpitaux de Paris, Hôpital Necker, Paris, France; 7 Paediatric Haematology, Evelina London Children’s Hospital, Guy’s and St Thomas’ NHS Foundation Trust, London, UK; 8 European Network of Rare Hematological Diseases, the ERN-EuroBloodNetPediatric Hematology Oncology Unit, Department of Women’s and Child’s Health, University of Padova, Padua, Italy

**Keywords:** Sickle cell disease, Patient education, Patient therapeutic education, Public patients involvement in Research, Patients workshop, International congress

## Abstract

**Background:**

Sickle cell disease (SCD) is an inherited chronic life-threatening disorder with increasing prevalence in Europe. People living with SCD in Europe mainly belong to vulnerable minorities, have a lower level of health education and suffer from isolation compared to those living with other chronic conditions. As a result, SCD patients are much less likely to partner in the design of research related to their condition and are limited in their ability to influence the research agenda. Aiming to increase the influence of patient voice in the development of SCD-related research, we set out to develop patient centered actions in the frame of International Scientific Conferences in collaboration with the ERN-EuroBloodNet, Oxford Blood Group, Annual Sickle Cell Disease and Thalassaemia Conference (ASCAT), the European Hematology Association and the British Society of Hematology.

**Results:**

Two events were organized: a one-day research prioritization workshop and a series of education sessions based on topics chosen by SCD patients and their families. Methodology and outcomes were analyzed in terms of influence on scientific, medical and patient communities.

**Conclusion:**

The ERN-EuroBloodNet workshops with patients at annual ASCAT conferences have provided an opportunity to enhance patient experience and empowerment in SCD in Europe, producing benefits for patients, caregivers, patient associations and health professionals. Future work should focus on delivering the research questions identified at this workshop and the opportunities to share information for patient education.

## Background

Sickle cell disease (SCD) is an inherited chronic life-threatening disorder resulting from the presence of a structurally abnormal haemoglobin known as haemoglobin S (HbS). This results in numerous complications including painful vaso-occlusion, vasculopathies, haemolytic anaemia, bacterial infections and strokes in adults and children. Long-term complications accumulate as SCD patients get older and life expectancy is reduced [1–3].

SCD is mostly prevalent in sub-Saharan Africa [4–6], where it leads to disability and premature death, even in societies with universal healthcare. SCD prevalence is increasing in other geographical areas due to global population movements [4]^;^ [7–11]. The World Health Organization has recognized this haematological condition as a public health area for which a global set of interventions are required, including development of new drugs and curative procedures, universal newborn screening, genetic counselling, medical education, clinical research, the development of clinical practice guidelines, access to highly specialized procedures, as well as patient advocacy and awareness campaigns [12, 13].

This increased prevalence compounded with improved life expectancy in countries where healthcare systems support the management of chronic conditions has led to an important economic and health burden. Nevertheless, the implementation of specific strategies at national level to tackle this condition is variable across EU members [12, 13].

People living with SCD in Europe mainly belong to ethnic and social minorities have a lower level of health education and are more likely to suffer from isolation, to have a poorer awareness of their condition and their needs could be invisible, as their voices are not heard. Therefore, they have difficulties in advocating to the public or engaging with public institutions and authorities [14]. This situation may be less acute in the UK or France where the prevalence of SCD is higher [15, 16], and many services are available at national level such as specialized health care providers, patient associations, and educational events. However, people of black and minority ethnic backgrounds in Europe are subject to endemic health inequalities and are less likely to participate in research [12, 13]^;^ [16, 17]. Within communities, the enduring stigmatization of SCD – often a legacy inherited from countries of origin – silences individuals with the condition and constrains engagement between patients and health care professionals [18, 19]. As a result, people with SCD are much less informed about patient therapeutic education, they report mistrust of large institutions including health care institutions and are much less likely to partner in the design of health care and research related to their condition, decreasing their ability to influence the health planning and the research agenda.

Improving the involvement of patients without an established voice in the development of research related to SCD may help to ensure that future research meets the needs of patients and improves access to educational opportunities. In addition, empowered patients who have been invited as equal partners with clinicians and scientists in discussions around the research agenda are more likely to become effective leaders in their patient communities and to contribute to service development and patient education [20] as well as help increase recruitment to established and future research studies.

Some initiatives have helped improve the visibility of this topic, such as the European Haematology Association (EHA) and the British Society for Haematology (BSH) highlighting these conditions as requiring additional focus. In addition, through the creation of the European Reference Networks (ERNs), the European Union has provided the requirements for tackling SCD at a European Level and increasing patients’ awareness across European Member States [21]. The European SCD educational plan is endorsed by the ERN-EuroBloodNet (www.eurobloodnet.eu), the ERN on Rare Haematological Diseases. The ERN-EuroBloodNet is well placed to coordinate the collaboration between people living with SCD and clinical and scientific teams, through interactions with experts, EURORDIS, Red Blood Cell European Patient Advocacy Group (ePAG) and European, National or Local patient groups.

In a collaboration between ERN-EuroBloodNet, Oxford Blood Group, Annual Sickle Cell Disease and Thalassaemia Conference (ASCAT), EHA and BSH we set out to develop both a research prioritization workshop with people living with SCD, as well as a series of education sessions based on topics chosen by patients. In this manuscript which includes a dedicated analysis of the SCD Prioritization Research Workshop in 2019 and of the online SCD patient education session in 2020, we report the methodology used for both projects, as well as the impact this has had on the scientific, medical, and patient communities.

## Methods

To structure a European patient education program in SCD, a meeting with ERN-EuroBloodNet SCD experts was held at the EHA 2019 Congress. It was decided that a patient forum would be created that would offer education as part of the ASCAT Conference program.

The ERN-EuroBloodNet SCD Working Group and ASCAT Steering Committees organized the SCD patient education training at ASCAT, with the following aims:

- Establish a European forum of SCD patients and carers whose objectives are to advocate for reducing isolation, identifying burdens regarding living with SCD, share best practices, patients’ rights, support and education for patients.
- Assess the effects and the impact of educational training on people living with SCD at a European level.
- Promote Public and Patient Involvement (PPI) in research, peer review and care, strengthening the relationship between physicians and patients.
- Establish strategies to increase attendance of SCD patients and their families at educational meetings using interactive and didactic formats.
- Make the patients’ perspective visible to the scientific community during international scientific conferences.

The overall design of this project was to embed the patient sessions in the ASCAT conference, an established international scientific congress focusing on SCD. For ASCAT 2019 and ASCAT 2020 the project team developed two innovative programmes with a central theme of patient and clinician education through mutual interaction. For both years, SCD patients were identified through ERN-EuroBloodNet representatives in collaboration with National SCD Networks, ASCAT members and EHA. Workshops were facilitated by the patient engagement group set up by the Oxford Blood Group, whilst ERN-EuroBloodNet, EHA, and BSH provided support for the educational sessions.

### Research prioritization workshop (ASCAT 2019)

**Fig.1:**
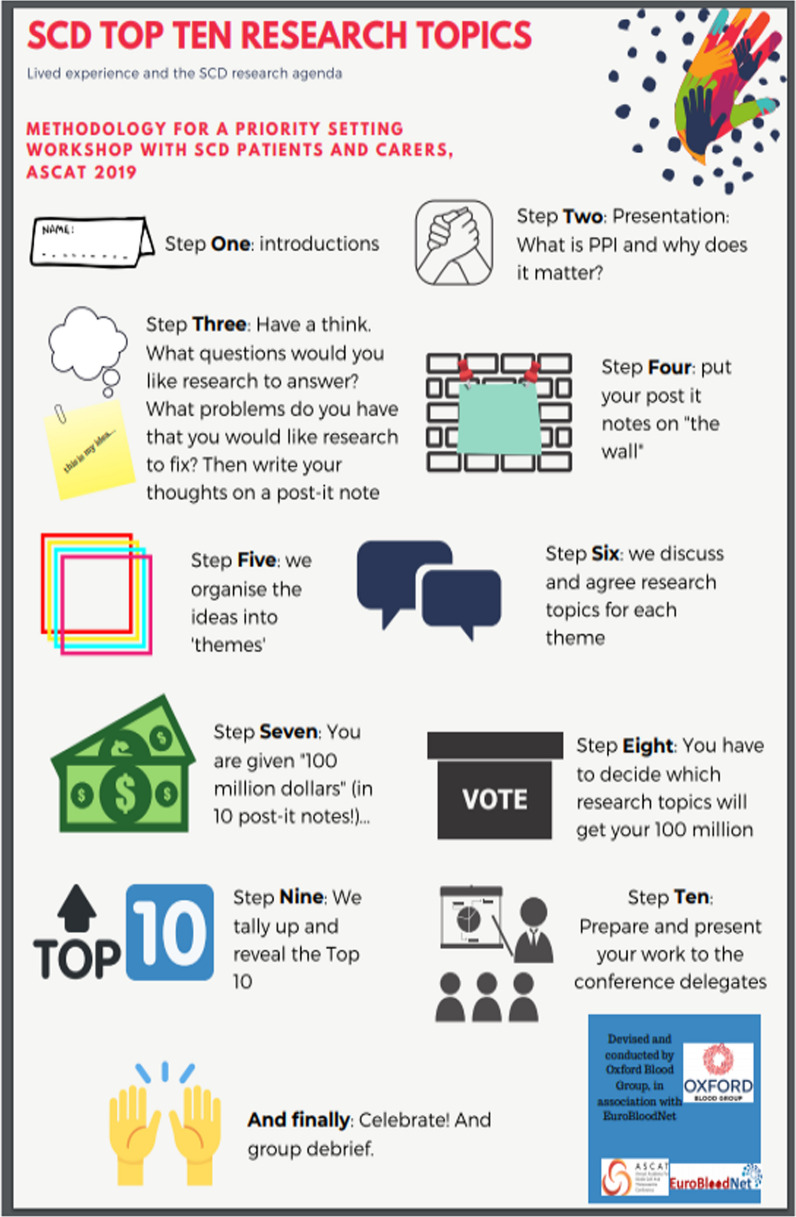
Abridged version of methodology for priority setting about Patient Public Involvement in Research (PPI)

One established methodology for research priority setting is the James Lind Alliance (JLA) Priority Setting Partnership (PSP) model [22, 23]. This identifies unanswered questions in specific health conditions from all relevant stakeholders, and then prioritizes them to produce a ‘top ten’ research topics which reflects the priorities of both the patients and the medical/scientific community. This involves a process of initial consultation to identify unanswered research questions. This long list is gradually refined, widelegates agreeing and prioritizing at collaborative workshops. While the outcomes are representative of the views of all stakeholders, the drawbacks are cost and time, with the process taking over a year to complete. We proposed an abridged version of this process, with twenty-eight delegates raising issues on the day, thematically arranging them, and then formulating questions as a group (Fig. 1). We decided that delegates would be asked to vote on these issues, as if they held a fund to be allocated to actual research projects. In our one-day condensed approach, only the views of the patients were sought, while medical professionals were not invited. The workshop was facilitated by a clinician who has previously led a JLA PSP, a SCD specialist nurse and a patient experience specialist. In addition, a clinical psychologist for people with SCD was present to provide some safety net should distress be expressed. (Fig. 1).

### Co-designed patient education sessions (ASCAT 2020)

For the 2020 patient education program, topics were identified by ERN-EuroBloodNet Coordination Team, ERN-EuroBloodNet members experts of SCD and representatives of ASCAT steering committee organizing the patient session (including ERN-EuroBloodNet, Oxford Blood Group, ASCAT, the EHA and the BSH). The following topics were selected: living with SCD during the COVID19 pandemic, participation of SCD patients in research, peer review of haemoglobinopathy services in the UK, and three expert panels: one “meet the experts” and two “meet the patients” including living with SCD as a paediatric patient. For the first panel five physician experts delivered lectures on the top five topics identified on a patient survey conducted by the ERN-EuroBloodNet Survey for prioritizing SCD topics to be addressed by education. It gathered 24 answers expressed by people living SCD from: Spain (8), France (5) Italy (3), Portugal (1), Cyprus (1), USA (1), and Belgium (1), NA (4) by number of contributors per country. Topics were; newborn and infant SCD screening, neurological complications in SCD adult people, quality of life in SCD, survival, infertility and other complications of bone marrow transplantation and new therapies for SCD. Additionally, we organized an expert panel of adult patients to answer questions from teenage patients and an expert panel of patients to answer questions from physicians. For each panel, a specific survey was conducted among registered participants to the ASCAT 2020 SCD Patient Educational Session in order to gather questions to be addressed by the panel. The joint effects of identifying topics among patients community and having a peer balanced panel made of patients and physicians guaranteed a patient-based session. So, the topics were chosen based in part on the top 10 topics from SCD Research Prioritization Workshop in ASCAT 2019 and was supplemented by an ERN-EuroBloodNet SCD Patients’ educational needs survey, (see Table 1).

**Table 1:** Results from ERN-EuroBloodNet Education Survey Needs for patients’ representatives with SCD in order of priority

1	New therapies for SCD
2	Adult patients quality of life
3	BMT: survival infertility and other complications
4	Neurological complications in SCD
5	Neonatal screening
6	Gestational risk
7	SCD and immune disease
8	Genetic counselling
9	Hydroxyurea and fertility
10	Polyuria and Enuresis: kidney damages
11	Priapism

For the 2020 session, the COVID19 pandemic resulted in the conversion of the conference to an online one, allowing a much wider participation by patients and carers who would otherwise not have been able to travel to Central London due to work, health or childcare issues. Indeed, forty-eight people living with SCD registered to the educational program. The Educational Online Session was fully recorded following signed, informed consent, in line with the General Data Protection Regulation (GDPR). It also gave patients access to the entire ASCAT Congress event.

One of the central themes of the development of these sessions was that they should not happen merely in parallel to the congress, but that patients should be invited to present the result of their workshops back to the plenary sessions of the congress. To this effect, a feedback session entirely led by the patients was scheduled into the main conference agenda, ensuring that the medical and scientific audience would listen to the patients.

## Results

### Research prioritization workshop at ASCAT 2019

**Fig. 2:**
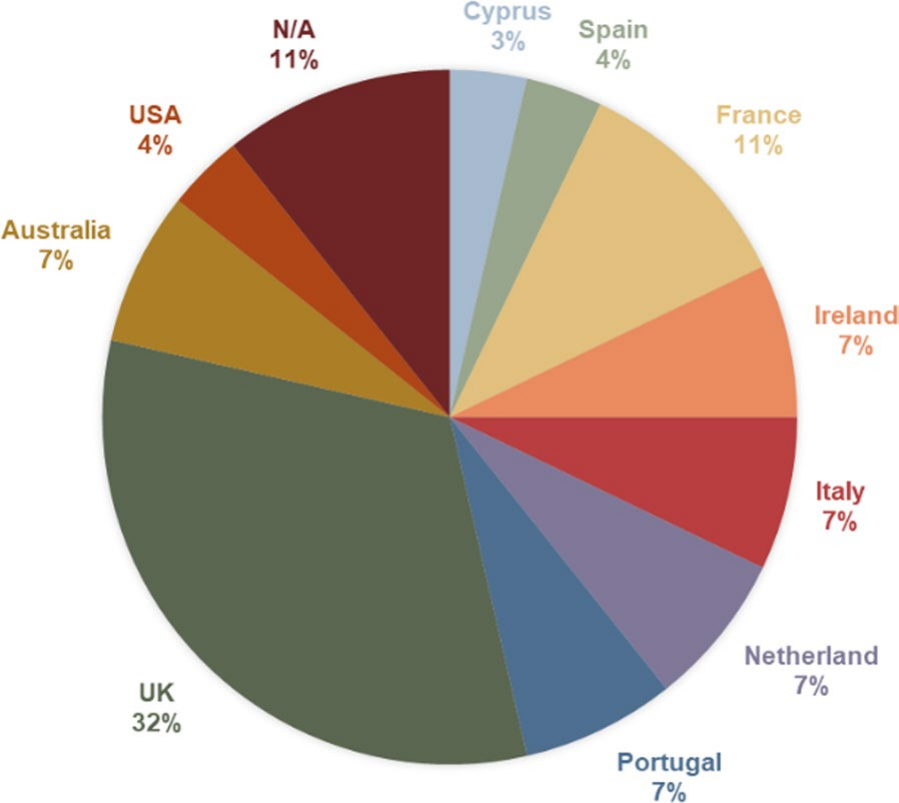
Distribution of participants by country of residence

A group of 36 people from 10 countries were invited to ASCAT 2019 to create a patient forum to share experiences, build cross-border partnerships and discuss good practice for local patient support groups. This group was made of 28 people living with SCD and 8 health professionals or ERN-EuroBloodNet representatives (Fig. 2).

#### Top ten research priorities

**Fig. 3:**
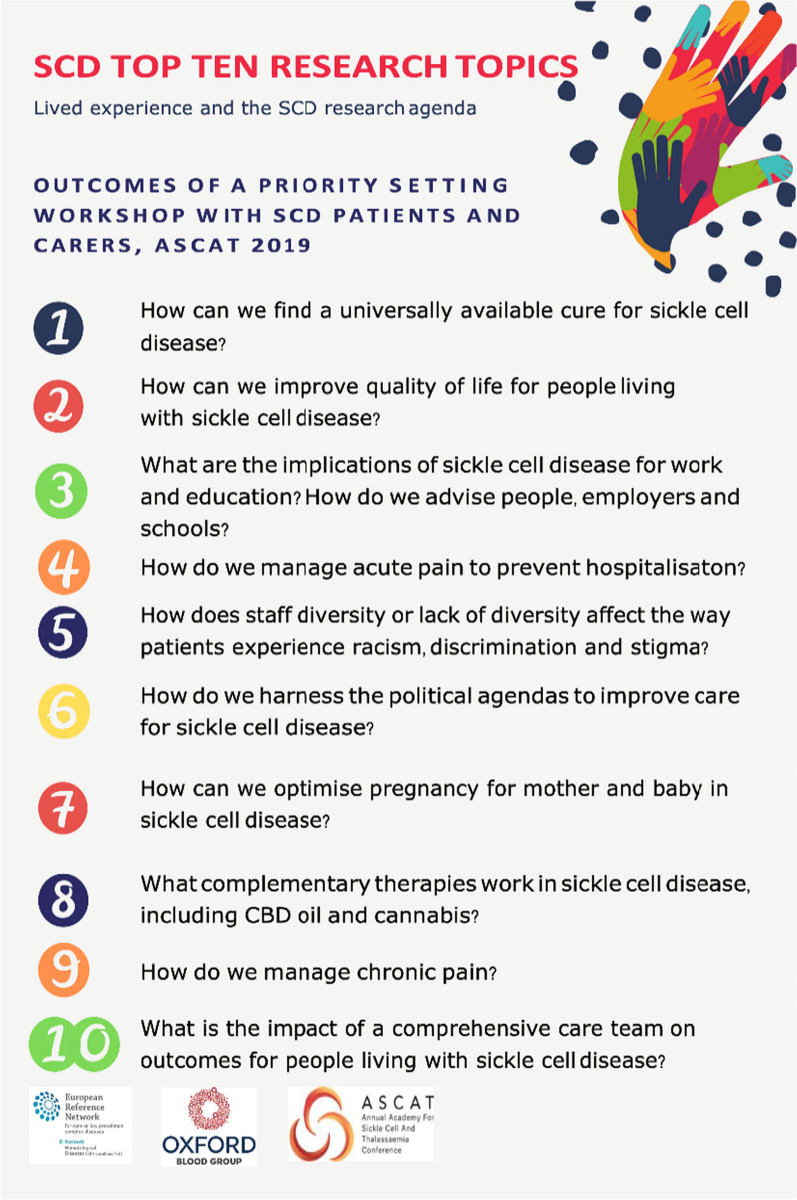
Outcome of a priority setting workshop: SCD Top ten research topics

At the workshop, patients were given specific training on the basic principles of Patient Public Involvement in Research. They were then asked to draw on their own experience of SCD, to write down issues that they felt demanded greater attention from research- a single word, an experience, or a fully formed research question. This led to a joint construction of a number of issues grouped together into separate themes. Using a co-development process, these themes were then summarized into 42 unique research questions- requiring a consensus approach between patients with differing life experiences and with access to variable national models of care. Patients were then asked to rank their individual “Top 10” research question. This was done by providing each patient with a fictional “100 million pounds” and asked to consider the question “if this was your money, which research projects would you fund?” The “Top 10” were selected by highest number of votes (Fig. 1). Finally, the group created a summary of the methodology, results, and presented it to the ASCAT conference in the plenary session. (The Top 10 Research Topics which were devised and selected by the group are shown in Fig. 3). Hearing from the people living SCD perspective on Public patients involvement in Research at the plenary of a scientific congress is a novel and optimal approach to guide healthcare professionals in consideringpatients’ viewpoint when designing research studies.

While the top priority for patients is to find a universally available cure, it is of note that quality of life as well as social and political issues were high on the agenda, and represent topics that the research community should focus on in addition to biological issues traditionally funded.

#### Patient satisfaction

Assessment of patient satisfaction reported the following results; all participants felt the experience was as expected or better, with 40% stating it was “much better than expected”. 80% of patients felt they had gained “a lot” from the experience. 75% of patients felt enough attention had been paid to those for whom English was not their first language. Most striking however was the free text feedback where patients were able to express how valuable they had found the experience of meeting others with the same condition from different countries, sharing lived experiences, and having the opportunity to discuss the issues they felt still need research attention. The ability to present the outcomes of their workshop to the plenary session was also powerful, both for the patients and for the clinicians and researchers who were being introduced to a new model of patient engagement. Participants from ASCAT 2019 provided high feedback scores for the event and appreciated the opportunity to voice their research priorities. Click to see patients’ testimony videos in the ERN-EuroBloodNet’s Education YouTube channel (cf. video [25] https://youtu.be/RyjCL311DYw) and patients’ presentation with workshop outcomes in the Supplementary data furnished in the Annex ASCAT 2019.

### SCD patients educational session at ASCAT 2020

**Fig. 4:**
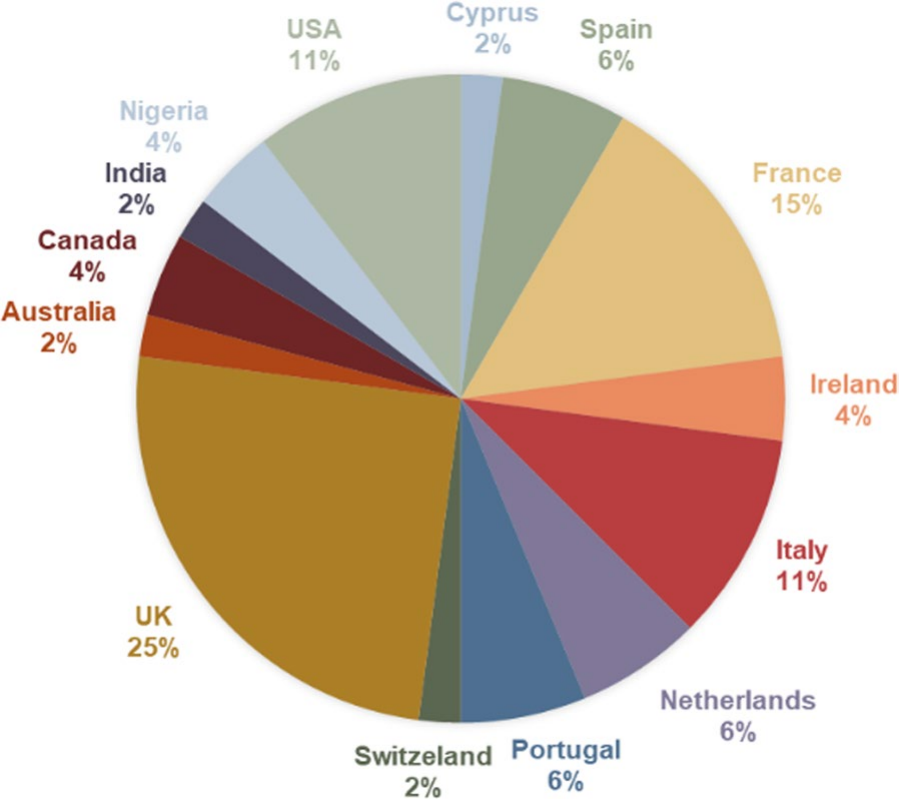
Distribution of participants by country of living

Forty-eight patients and parents registered for the event, which was conducted entirely online. The event was also supported by 20 health professionals, mostly from European countries. (See Fig. 4 for the countries represented among the patients and parents). The creation of such a group resulted in improved experience of the conference and patients felt less isolated by sharing opinions, experience and advocating at a European and global level. In addition, it provided an occasion for learning about scientific outcomes in SCD, clinical patient management, healthcare quality assurance processes. The increase in the number of participants confirms a progression in training a motivated groups of patients and their families by having common goal across Europe to improve outcomes forpeople living with SCD.

The 2020 session was dedicated to enhancing health literacy among patients and it was focused on the topics chosen by patients themselves. Each education session was dual-moderated by one physician and one patient representative. Their role was to present the topic, gather key messages and collect questions raised by attendees in the platform's chat. For some topics, pre-recorded sessions of patient testimonies were shared. To shape the framework of each specific session, questions from clinicians and young SCD patients to be answered by expert patients were collected ahead of time (see Tables 2 and 3 respectively). This is an innovative approach to education where clinicians learned from the expertise of patients through an interactive Q&A session and teenage patients had the opportunity to learn from adult patients about their condition and their approach to its management.

**Table 2:** Questions from clinicians and young SCD patients to expert patients

1	As someone living with SCD when you have a painful crisis what do you want your doctor to ask you?
2	What feature in any new therapy would make you agree to adopt it?
3	What better ways are there for doctors to communicate with you?
4	What is the right term: ‘people with SCD’ or ‘patient with SCD’
5	How can we tackle racism and stigma from healthcare providers?
6	Do you feel you have enough information if you got COVID19 and if not what can be done?

**Table 3:** Questions from teenage SCD patients to adult patients

1	If you got the chance to change 3 things in your journey till your mature age, what would you’ve changed?
2	Will I be able to live normal like other kids and be strong to do anything like other normal kids?
3	Will I be on medication throughout my lifetime even if I oft for bone marrow transplantations
4	How does it affect relationships?
5	When I’m older can I still enjoy fun activities with my friends? I want to get piercing but I’m always told not to and I feel like I’m missing out
6	Does the sickle cell disease go at a certain age or not?
7	What are the health challenges on older people in these different areas: Employment, sex, hospitalisation, vacation by air?

Feedback survey from participants at ASCAT 2020 was excellent, with 93% participants scoring 4 or 5 out of 5 in overall rating for the event. 89% gave high scores on the topics discussed, > 90% approved the patient and clinician jointly moderated sessions with high ratings and 95% preferred the online format of the programme. This demonstrated a significant educational impact on participants. Patient testimony videos can be viewed on the ERN-EuroBloodNet’s Education YouTube channel (cf. video [26] https://youtube.com/playlist?list=PLpldFGPsMHrmumLznkvX-OOjcADYMgY1K) and patients’ presentation with workshop outcomes in the Supplementary data furnished in Annex ASCAT 2020.

New educational needs were identified during the whole session, as presented at the conference plenary session and video testimonies of lived experience of two patients. SCD patients and parents indicated their desire to have access to information on mental health, pregnancy, and side effects of bone marrow transplantation.

The ASCAT 2020 education session highlighted how a virtual platform can result in good participation and meaningful interactions with other patients and clinicians. A post-event survey also indicated that the online format resulted in less cost, avoiding the physical inconvenience of travel which often leads to fatigue, time needed off work and arranging childcare and was the choice of future event formats by patients.

## Discussion

Our innovative patient-centred approach of embedding both research prioritization workshops in 2019 and patient-driven education sessions in 2020 both within an international conference of SCD professional experts has produced accessible educational resources for patients, caregivers, patient associations and health professionals.

Seventy-six participants (twenty-eight in 2019 and forty-eight in 2020) learned about the latest advances in the field of haemoglobinopathies, the best management of the disease, and patient involvement in research and healthcare quality assurance processes. Involving patients in research and care of rare haematological conditions such as SCD is difficult and not often done, and this conference was an effective means of improving knowledge in both patients and physicians, and empowerment of the former as expressed by the patients’ satisfaction, by the higher number of participants in the second year. This was also because it was offered an educational online program, meaning an easier access to trainings in terms of reducing burdens to fatigue, family and work time.

The first relevant result obtained from the experience of ASCAT 2019 and the research prioritization workshop for public patient involvement in research was that the focus of investigation from patients’ point of view differs from the health professionals’ one. Although it is not possible to compare the top 10 priorities to a full JLA PSP list, as none has been carried out in SCD, the identification of priorities for SCD from a patient’s perspective that is slightly different from the priorities that health professionals bring forward needs to be underlined. Patients’ point of view on priorities for research was given to social and political aspects like quality of life, stigma or racism; or even complementary therapy. This compares to how research funds are currently allocated to biological aspects of SCD. In fact, recent data coming from the international Sickle Cell World Assessment Survey (SWAY) [24] shows that symptom perception, emotional impact and priority treatment goals are not perceived equally in health care staff and patients. Prioritization of health care interventions and research projects should take this information into consideration in future planning in which patients should have a more significant role.

In what we believe were the first meetings of European patients with SCD, we show that there is common ground of unmet need for patients with SCD across the EU. As the European health policy on rare disorders (RD) moves to a wider share of health data, increase of cross-border health care, patients and health care dialogue and empowerment is crucial to help shape actions at a European Level [21].

Moreover, the outcome of these events has shown that people living with SCD are capable of articulating their views given the right platform. It is a challenge if these recommendations do not lead to tangible increase in research funding channeled towards projects that address the issues identified by patients.

SCD patients in Europe often belong to socially and economically vulnerable groups and/or recent residents due to population movements. This leads to the lack of SCD European Organizations or European representatives as identified by ERN-Eurobloodnet early in its creation in 2017. There is a minor representation of patient advocates for this condition compared to other rare anemias such as Thalassemia, as identified by the ERN-EuroBloodNet early in its creation in 2017. The participation of seventy-six SCD patients or carers in these two European meetings with a dedicated space to be trained and express themselves may contribute in reducing the stigma of this chronic hematological disorder [18, 19]. This experience has also created a motivated group of patients that can advocate at both the European and global levels. This forum talked about the needs to be improved for patients with SCD all over the world. Their thoughts could be read in the SCD Patients Priority Blog (webpage^27^ https://sicklecellpatientpriorities.wordpress.com) developed following ASCAT 2019 to give a public space where people living with SCD could express themselves. With this model of health education, it has been shown that patient involvement is possible and meaningful, it makes a difference to patient groups, and it strengthens the relationship among physicians and patients. Finally, hearing patients’ perspective during an international scientific conference has been highly appreciated from both health professionals and patients alike. Following these events, a patient forum was created and has resulted in increasing participation from patients in successive conferences.

Some challenges were noted during this experience. Patients had different levels of health education and disease awareness, making it harder to provide content that was suitable for all. In addition, as the whole conference was held in English, posing an additional challenge for those in whom English was not a first language and more effort will be needed to find ways to overcome language barriers and increase patients’ involvement. The virtual format has been appreciated by patients more than the face-to-face format and may be continued in future.

## Conclusion

The ERN-EuroBloodNet workshops with patients at ASCAT conferences have provided an opportunity to enhance patient experience and empowerment in SCD in Europe and identify areas of priorities for patients and their caregivers. In addition, similar educational programs should be enhanced and expanded to SCD patients in other countries than European Member States. Future work should focus on delivering the research questions posed at this workshop and continue to create credible opportunities to share information for patient education.

## Data Availability

Not applicable.
